# Approaching a nationwide registry: analyzing big data in patients with heart failure

**DOI:** 10.55730/1300-0144.5931

**Published:** 2024-05-07

**Authors:** Tuğçe ÇÖLLÜOĞLU, Anıl ŞAHİN, Ahmet ÇELİK, Emine Arzu KANIK

**Affiliations:** 1 Department of Cardiology, Faculty of Medicine, Karabük University, Karabük, Turkiye; 2 Department of Cardiology, Faculty of Medicine, Sivas Cumhuriyet University, Sivas, Turkiye; 3 Department of Cardiology, Faculty of Medicine, Mersin University, Mersin, Turkiye; 4 Department of Biostatistics and Medical Informatics, Faculty of Medicine, Mersin University, Mersin, Turkiye

**Keywords:** Heart failure, big data, real-world data, nationwide study, biostatistic

## Abstract

**Background/aim:**

Randomized controlled trials usually lack generabilizity to real-world context. Real-world data, enabled by the use of big data analysis, serve as a connection between the results of trials and the implementation of findings in clinical practice. Nevertheless, using big data in the healthcare has difficulties such as ensuring data quality and consistency. This article aimed to examine the challenges in accessing and utilizing healthcare big data for heart failure (HF) research, drawing from experiences in creating a nationwide HF registry in Türkiye.

**Materials and methods:**

We established a team including cardiologists, HF specialists, biostatistics experts, and data analysts. We searched certain key words related to HF, including heart failure, nationwide study, epidemiology, incidence, prevalence, outcomes, comorbidities, medical therapy, and device therapy. We followed each step of the STROBE guidelines for the preparation of a nationwide study. We obtained big data for the TRends-HF trial from the National Healthcare Data System. For the purpose of obtaining big data, we screened 85,279,553 healthcare records of Turkish citizens between January 1, 2016 and December 31, 2022.

**Results:**

We created a study cohort with the use of ICD-10 codes by cross-checking HF medication (n = 2,722,151). Concurrent comorbid conditions were determined using ICD-10 codes. All medications and procedures were screened according to ATC codes and SUT codes, respectively. Variables were placed in different columns. We employed SPSS 29.0, MedCalc, and E-PICOS statistical programs for statistical analysis. Phyton-based codes were created to analyze data that was unsuitable for interpretation by conventional statistical programs. We have no missing data for categorical variables. There was missing data for certain continuous variables. Propensity score matching analysis was employed to establish similarity among the studied groups, particularly when investigating treatment effects.

**Conclusion:**

To accurately identify patients with HF using ICD-10 codes from big data and provide precise information, it is necessary to establish additional specific criteria for HF and use different statistical programs by experts for correctly analyzing big data.

## 1. Introduction

Healthcare data, including information about patients with heart failure (HF), is highly sensitive and subject to stringent privacy and security regulations like the Health Insurance Portability and Accountability Act (HIPAA) in the USA or General Data Protection Regulation (GDPR) in Europe, e-Pulse portal (e-Nabız) in Türkiye [1,2]. Compliance with these regulations is imperative to ensure the confidentiality and security of patient data, adding layers of complexity to data access procedures.

Big data has been defined as the aggregation of vast amounts of digital information from diverse sources, holds immense potential to reshape the landscape of healthcare by enabling comprehensive data analysis and generating actionable insights [3]. In the realm of healthcare, big data encompasses a wide range of sources, including electronic health records, public health data, clinical research findings, and real-world evidence [3,4]. These various sources converge to form a comprehensive repository of health-related information, facilitating the exploration of trends, patterns, and correlations across populations and disease states [5]. However, alongside its considerable benefits, the widespread adoption of big data in healthcare necessitates careful consideration of data accuracy. Researchers face several obstacles in acquiring reliable datasets, grappling with issues of data quality, completeness, and accuracy stemming from the diversity of data sources [6]. The reliability and validity of big data are paramount for meaningful research and analysis, necessitating specialized expertise in data management, analysis, and security. Ensuring big data consistency across various sources remains a critical challenge in utilizing healthcare data for scientific research and clinical decision-making.

**Figure 1:**
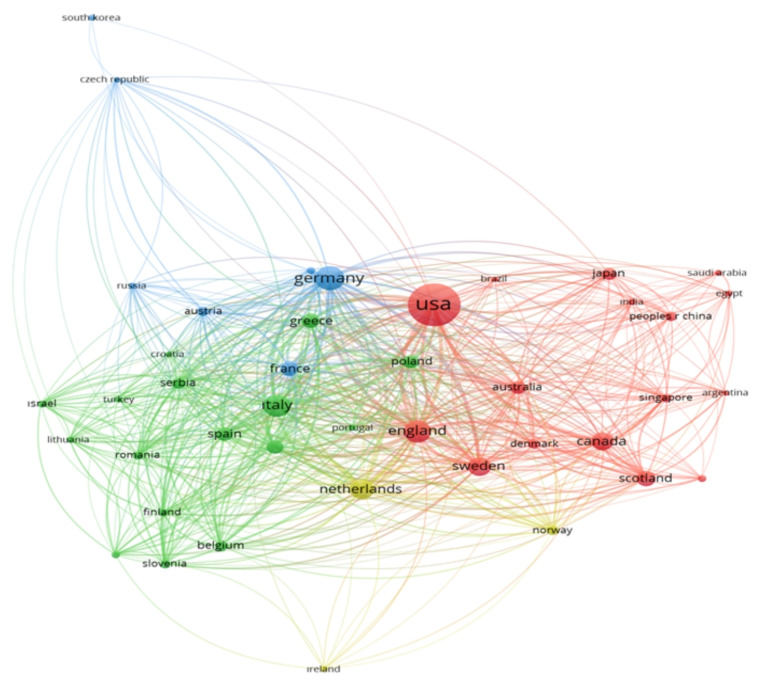
Countries that have conducted big data analysis on heart failure.

Heart failure is a prominent area of interest for big data research within cardiovascular studies. Because, HF is associated with multiple underlying etiologies, risk factors, and comorbidities. In addition, HF exhibits a progressive nature of the disease course [7]. The use of big data in HF research is evident through its diverse origins, utilizing numerous countries sources as illustrated in Figure 1. These sources cover a broad spectrum, ranging from electronic health records and medical imaging to genomics and wearable devices [5]. By integrating data from such varied streams, researchers gain a comprehensive understanding of HF pathophysiology and progression, paving the way for enhanced diagnostic and therapeutic strategies.

This article aims to explore the several challenges encountered in accessing healthcare big data for cardiovascular research, particularly in the context of HF. By elucidating the complexities surrounding big data access, quality assurance, and resource allocation, this study seeks to provide insights into overcoming these barriers based on the our experiences when creating Trends in heart failure between 2016 and 2022 in Türkiye (TRends-HF) study which is a Nationwide registry for HF from Turkiye [8].

## 2. Materials and methods

**Figure 2:**
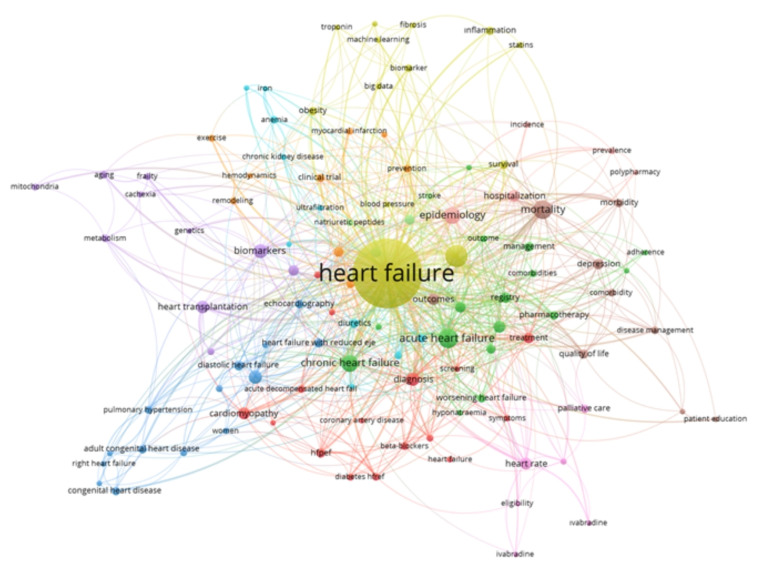
Key words commonly used for heart failure in nationwide studies.

The TRends-HF study represents a comprehensive big data investigation conducted using a national registry database in the field of HF [8]. The study design involved the formal appointment of a multidisciplinary team comprising cardiologists, biostatistics specialists, and data analysts to oversee all aspects of the study. Prior to commencing data collection, we delved into various databases such as Pubmed, MEDLINE, EMBASE, Web of Science, and Cochrane Central Register of Controlled Trials, and scholarly sources to identify relevant clinical trials related to HF, gathering insights into methodologies, outcomes, and gaps in knowledge. Figure 2 shows the key words frequently used in nationwide studies on heart failure. We used the keywords as follows: heart failure, nationwide study, epidemiology, incidence, prevalence, outcomes, comorbidities, medical therapy, and device therapy. And then, we identified suitable trials from the wide range of research available. We meticulously screened studies based on criteria such as methodology and relevance to epidemiological inquiry. Adhering to the STROBE (Strengthening the Reporting of Observational Studies in Epidemiology) guideline was significant to ensuring the robustness of our observational trial [9]. We followed each step of the registry preparation process in STROBE guideline to enhance the quality, validity, and transparency of nationwide registry on HF.

The Ministry of Health of Türkiye approved the design of the TRends-HF study prior to data collection and statistical analysis with the approval number 95641342-020.

## 3. Results

Validating the diagnosis of HF and comorbidities based on ICD-10 codes, which were obtained from the Nationwide Healthcare database of Türkiye. National Healthcare database has the complete healthcare information of 85,279,553 Turkish citizens. Each individual in the National Healthcare database is assigned a unique identification number consisting of 11 digits and appeared only once in the dataset. During the process of retrieving information from the database, encrypted identification numbers were assigned to each patient in order to safeguard their privacy. Access to personal data, including patients’ identity information and contact details, has been limited. The diagnosis of HF according to ICD-10 codes was confirmed by cross-checking HF medications. If the patient was prescribed at least one medication for HF, the patient was diagnosed with HF. With this definition, it was found that a total of 2,722,151 patients were diagnosed with HF between 2016 and 2022. The identification of comorbid conditions and underlying etiologies was facilitated through the use of ICD-10 codes. The Anatomical Therapeutic Chemical (ATC) classification system was employed for the classification of all medications utilized within the study cohort. Procedures and diagnostic tests conducted within the study population were documented and coded according to the Health Application Communiqué (SUT) codes specified in the Turkish Republic Healthcare Implementation Communiqué. Mortality data were obtained from the Death Notification System of the General Directorate of Health Information Systems.

In our study, variables were obtained from the National Electronic Database. Variables were placed in different columns. These variables encompassed age, date of birth, sex, date of initial diagnosis, healthcare institution where the diagnosis was made, distribution of patients with HF based on cities and geographical regions, distribution of patients with HF based on the socioeconomic status of the provinces, accompanying comorbid conditions, medication information according to the ATC code, tests and procedures according to the SUT codes, date of hospital admission, types of hospital admission, length of hospital stay, date of death, and the duration from diagnosis to death. Laboratory variables such as BNP, NT-proBNP, albumin, creatinine, estimated glomerular filtration rate, Na^+^, K^+^, Ca^++^, uric acid, triglycerides, LDL-C, hemoglobin, ferritin, and HbA1c also added through the National Electronic Database.

The statistical analysis was conducted to examine trends in HF incidence, prevalence, concurrent comorbid conditions, medications, and outcomes in all age groups (0–110 years) during 6 years based on socioeconomic status. For all statistical analyses, different statistical programs, including SPSS 29.0 software (IBM Corp., Armonk NY, USA), MedCalc 9.3.9.0, and E-PICOS AI (MediCres, NY), were used. When individual computers and typical statistical programs were not enough to read and analyze data on a large-scale, Phyton-based codes were specifically developed as solutions. No data were deleted, and no new information were added to the missing data. Except laboratory data, there were no missing data in categorical variables. The absence of data on laboratory variables was associated with techniques that had not been completely defined for their application in HF. Median (IQR) interpretation was preferred for standardizing laboratory parameters. Patients’ follow-up and last follow-up were recorded as dates, and then dates were converted to time units. Given the size of the data, propensity score matching was conducted to ensure similarity among the studied groups, particularly when investigating treatment effects.

## 4. Discussion

Big data has substantial advantages in HF research by allowing access to large volumes of diverse data sources. This extensive data pool enhances the scope and depth of HF research. However, for accurately solving big data, there needs to be specialized expertise in cardiologists, biostatistics specialists, and data analysts. In the TRends-HF study, before creating this study, we first assembled a heart failure team consisting of training cardiologists, heart failure specialists, biostatistics specialists, and data analysts. All members of the team consisted of individuals who were highly specialized in the field. This collaborative endeavor aimed to develop a robust framework for tracking and analyzing health data on a national scale. In addition, each member was familiar with computer systems. We think that this familiarity with information technology may facilitate a more natural understanding of electronic health records and health informatics within the framework of HF. This facilitation can be supported by the familiarity of many HF fellows with information technology. Notably, a significant number of HF fellows have background knowledge in biology, computer science, and bioinformatics-fundamental disciplines pivotal for integrating big data analytics into clinical practice and research endeavors [10]. Moreover, the active involvement of HF fellows in frontline patient care may put them in a position to identify clinical needs, thereby catalyzing the generation of needed research ideas with direct applicability to clinical practice.

Routinely collected healthcare data from electronic health records, such as primary care databases, registries, and administrative healthcare claims, is commonly used in epidemiological studies to assess the real-world effectiveness and safety of medications [11]. The guidelines have suggested employing STROBE, RECORD, CONSORT, and STARD statements for various types of studies. The STROBE statement has been recommended for observational studies [9]. The TRends-HF study was a nonrandomized, observational study that included collected data from the National Electronic Healthcare Database [8], throughout each stage of the TRends-HF study, we consistently used the STROBE check-list to ensure the presentation of data with high levels of trustworthiness, accountability, and openness. In addition, the STROBE check-list allowed us to maintain control over the establishment of TRends-HF. Finally, these statements enable readers to easily recognize the research topic, the employed technique, and the study findings, and to enhance the comprehensiveness of the study limitations [9, 11]. Furthermore, they can indirectly improve the research’s quality by highlighting the specific aspects that need to be considered throughout the design of the study [9]. In summary, our nationwide registry has been marked by meticulous attention to detail, adherence to STROBE guidelines, a commitment to producing reliable and impactful results in the field of HF epidemiology.

Since 2016, the Turkish Ministry of Health has started to regularly record all diagnoses using ICD-10 codes for every individual who presents to health institutions. The validity of ICD-10 codes was examined for several types of chronic diseases, including HF, in epidemiological trials consisting of big data from several countries [12–15]. The study conducted in France revealed that the positive predictive value (PPV) of ICD-10 code I50.x, as defined by European Society of Cardiology, was determined to be 88% [16]. Despite a high level of PPV, the sensitivity of ICD-10 codes for HF was found to be comparatively lower [16]. The low sensitivity of the ICD-10 code I50.x makes it unsuitable to rely solely on these codes in epidemiological studies that aim to investigate the incidence of prevalence of HF. Hence, it was essential to verify medication usage by cross-refernecing it with ICD-10 codes in order to precisely ascertain the prevalence and incidence of HF cases in our registry. Additionally, a crucial aspect of our registry involved establishing epidemiological data on HF in Türkiye for the first time. This required rigorous data collection, analysis, and interpretation to provide accurate insights into the burden of HF on our population. We dedicated considerable effort to researching strategies aimed at avoiding the overestimation or underestimation of the prevalence and incidence rates of HF. This involved critically evaluating potential biases, refining our methodologies, and employing statistical techniques to ensure the integrity of our findings.

Missing data is a prevalent issue in epidemiology, and participant dropouts can substantially reduce the sample size available for analysis in initially large cohorts. Missing data can result in bias and always lead to a decrease in efficiency [17]. There are different recommendations available. The literature reports that multiple imputations are not helpful when the missing data is less than 5% [18]. On the other hand, according to a specific online tutorial, a maximum upper threshold of 5% missing data is recommended for large datasets^1^. According to statistical guidelines, studies with more than 10% missing data are likely to have bias [19]. Especially, more than 40% of the missing data in important variables should be considered hypothesis-generating [20].

Analyzing big data requires sophisticated computational methods and expertise in data science. Interpreting complex algorithms and statistical models may be challenging for clinicians and researchers, leading to potential misinterpretation or overreliance on data-driven insights [21, 22]. To overcome this barrier, data scientists, computer scientists, health informatics specialists, and cardiologists had to engage in team science to harness the full potential of big data analytics in HF research. Therefore, we created a team. Beyond building our team, in the TRends-HF study, our approach to data analysis extended beyond traditional statistical programs such as SPSS, MedCalc, and E-PICOS to accommodate the complexities inherent in big data. While conventional statistical tools played a crucial role in many aspects of our statistical analysis, they were unable to interpret certain data. We specifically created Phyton-based codes to address this challenge, revealing the data and enabling efficient extraction, processing, and analysis of the vast array of data points. Therefore, Phyton-based codes led to an accurate examination of all relevant variables. By embracing innovative solutions like Phyton-based coding, we were able to overcome the limitations of standard statistical programs and unlock deeper insights into the trends and patterns observed within the HF patient population. This integrative approach may underscore the importance of flexibility and adaptability in data analysis methodologies, particularly when dealing with large-scale datasets, which are characteristics of big data research in cardiology.

## 5. Conclusion

To summarize, whereas big data has remarkable prospects for advancing cardiology research and improving patient care, it can also pose several problems and considerations that need to be resolved. Colloboration among researchers, clinicians, and data scientists is crucial for effectively using big data in the field of cardiology, while also addressing its limitations and assuring responsible use. By overcoming these challenges, big data has the potential to completely transform our comprehension and management of HF and other cardiovascular diseases.

## Footnotes

1 Data Science Plus (2015). Imputing missing data with R; MICE package [online]. Website https://datascienceplus.com/handling-missing-data-with-mice-package-a-simple-approach/ [accessed 04 October, 2015]
